# Genetic evidence for the interaction between *Bacillus anthracis*-encoded phage receptors and their cognate phage-encoded receptor binding proteins

**DOI:** 10.3389/fmicb.2023.1278791

**Published:** 2023-10-31

**Authors:** Samantha Forrest, Sarah Ton, Samantha L. Sholes, Sarah Harrison, Roger D. Plaut, Kathleen Verratti, Michael Wittekind, Elham Ettehadieh, Bryan Necciai, Shanmuga Sozhamannan, Sarah L. Grady

**Affiliations:** ^1^Johns Hopkins University Applied Physics Laboratory, Laurel, MD, United States; ^2^Division of Bacterial, Parasitic, and Allergenic Products, Center for Biologics Evaluation and Research, Food and Drug Administration, Silver Spring, MD, United States; ^3^Olympic Protein Technologies, Seattle, WA, United States; ^4^Joint Program Executive Office for Chemical, Biological, Radiological and Nuclear Defense (JPEO-CBRND), Joint Project Lead for CBRND Enabling Biotechnologies, Frederick, MD, United States; ^5^Joint Research and Development, Inc., Stafford, VA, United States

**Keywords:** *Bacillus anthracis*, phages, phage resistance, receptor binding proteins, bacterial receptors, S-layer, fluorescence detection

## Abstract

Bacteriophages such as γ and AP50c have been shown to infect strains of *Bacillus anthracis* with high specificity, and this feature has been exploited in the development of bacterial detection assays. To better understand the emergence of phage resistance, and thus the potential failure of such assays, it is important to identify the host and phage receptors necessary for attachment and entry. Using genetic approaches, the bacterial receptors of AP50c and γ have been identified as *sap* and GamR, respectively. A second AP50c-like phage, Wip1, also appears to use *sap* as a receptor. In parallel with this work, the cognate phage-encoded receptor binding proteins (RBPs) have also been identified (Gp14 for γ, P28 for AP50c, and P23 for Wip1); however, the strength of evidence supporting these protein–protein interactions varies, necessitating additional investigation. Here, we present genetic evidence further supporting the interaction between *sap* and the RBPs of AP50c and Wip1 using fluorescently tagged proteins and a panel of B. anthracis mutants. These results showed that the deletion of the *sap* gene, as well as the deletion of *csaB*, whose encoded protein anchors *sap* to the bacterial S-layer, resulted in the loss of RBP binding. Binding could then be rescued by expressing these genes in *trans*. We also found that the RBP of the γ-like prophage λBa03 relied on csaB activity for binding, possibly by a different mechanism. RBP_λBa03_ binding to *B. anthracis* cells was also unique in that it was not ablated by heat inactivation of vegetative cells, suggesting that its receptor is still functional following incubation at 98°C. These results extend our understanding of the diverse attachment and entry strategies used by *B. anthracis* phages, enabling future assay development.

## 1. Introduction

*Bacillus anthracis*, the causative agent of anthrax, is a spore-forming, Gram-positive bacterium that qualifies as a CDC Tier 1 select agent due to its potential use as a bioterrorism agent (Darling et al., 2002). Due to its impact on national biodefense, it is vital to have surveillance strategies in place that are rapid, reliable, and can be performed with limited equipment. (Bacterio)phages that specifically infect *B. anthracis* represent an appealing set of tools towards this end (Abshire et al., 2005; Sozhamannan et al., 2008; Schuch et al., 2010). Indeed, an engineered *lux* fusion reporter phage has been developed and tested for rapid detection of viable *B. anthracis* spores in environmental samples (Sharp et al., 2016). In addition to their potential utility in bacterial identification, there has also been a recent resurgence in use of phages as therapeutics due to the growing threat of multi-drug resistant bacteria (Aranaga et al., 2022). With all of these potential applications, it is critical to understand the genetic mechanisms driving phage resistance, allowing for the intelligent design of both therapeutic phage cocktails and diagnostic/surveillance tools.

An important step towards deciphering these resistance mechanisms is identifying phage receptor binding proteins (RBP) and their cognate bacterial surface receptor(s). The specificity of a phage for its target bacteria is attributed, at least in part, to the strength of this interaction, and receptors can be any of a diverse set of molecules, including proteins, (lipo)polysaccharides, and carbohydrate moieties (Rakhuba et al., 2010; Bertozzi Silva et al., 2016; Hyman and van Raaij, 2018). With respect to *B. anthracis*-specific phages, the bacterial protein GamR was identified as the bacterial receptor of phage γ almost two decades ago using genetic approaches (Davison et al., 2005). Identification of the bacterial receptors of other phages, including AP50c and Wip1, came later. These efforts started with whole genome sequence analysis of spontaneous AP50c-resistant *B. anthracis* mutants. Here, mutations in the *csaB* gene were found to result in phage resistance (Bishop-Lilly et al., 2012). As *csaB* is known to anchor the surface-array protein (*sap*) to the bacterial S-layer present on the *B. anthracis* vegetative cell surface (Mignot et al., 2002), it was postulated that *sap*, more appropriately the S-layer, could be the receptor for AP50c (Bishop-Lilly et al., 2012). S-layer, which itself provides structural integrity and stability to the cell wall (Fioravanti et al., 2022), was also hypothesized to be the bacterial receptor of Wip1 due to its genetic similarity to AP50c (Kan et al., 2013). More direct evidence in support of the AP50c-*sap* binding hypothesis was produced when incubation of purified *sap* protein with intact AP50c phage particles reduced the titer of free phage (Plaut et al., 2014). Ultimately, analysis of various transposon insertions and targeted mutants revealed that not only *sap* and *csaB,* but also the sporulation genes *spo0A*, *spo0B,* and *spo0F* all had roles in productive AP50c infection of *B. anthracis* cells (Plaut et al., 2014). While the direct roles of *sap* and *csaB* in the formation of the S-layer are clear, whether the sporulation genes play a direct or indirect role in either S-layer formation or phage attachment and infection is not clear at this time.

As the potential bacterial receptors of these phage were put forth, research was also conducted to identify their respective phage receptors. The RBP of Wip1 was identified as P23 (henceforth referred to as RBP_Wip1_) based on phage adsorption tests and immunofluorescence assays (Kan et al., 2013). The activity of P23 was found to require the translation of the downstream *p24* gene (Kan et al., 2013), which shares extensive sequence similarity to *p29* gene of AP50c (Braun et al., 2020). Based on its location immediately upstream of *p29*, along with a short amino acid sequence identity to the Wip1 P23 protein, the AP50c protein P28 (RBP_AP50c_) was then posited as its RBP (Braun et al., 2020). When the Wip1 *p23* and AP50c *p28* genes were then cloned into expression vectors with fluorescent protein genes, the resulting fusion proteins were shown to bind to *B. anthracis* cell surfaces (Braun et al., 2020). Together, this work supported the role of P23 and P28 in phage binding but did not establish their interaction with *sap*. Here, we describe a set of genetic and molecular experiments to further probe these phage-bacterial interactions and provide evidence that presence of *sap*, *csaB*, and the sporulation proteins Spo0A, Spo0B, and Spo0F are likely required for RBP_Wip1_ and RBP_AP50c_ binding to *sap* on *B. anthracis* cell surface.

In addition to prototypical phages, there have also been prophages identified in the chromosome of *B. anthracis*, including the γ-like prophage λBa03 (Sozhamannan et al., 2006). The genome of λBa03 encodes the hypothetical protein BA4079 (henceforth referred to as RBP_λBa03_) that shares significant amino acid sequence homology to γ phage encoded RBP Gp14, making it an ideal candidate for λBa03 RBP (Braun et al., 2020). Like RBP_AP50c_ and RBP_Wip1_, a domain of RBP_λBa03_ lacking the first 120 amino acids (designated as RBP_λ03Δ1-120_) has been shown to bind to the cell surface of vegetative *B. anthracis* bacterium, but little else is known about its bacterial target for binding. Here, we find that λBa03 represents a different paradigm than Wip1 and AP50c in that it requires *csaB* activity for binding, but that this phenotype appears to be unrelated to its role in anchoring *sap* to the S-layer. Intriguingly, RBP_λ03Δ1-120_ binding to *B. anthracis* also is maintained following heat treatment, meaning its receptor is not heat labile or protected from heat inactivation. These results underscore the significant diversity in strategies adopted by these phages and corresponding genetic requirements for attachment and entry during infection of their host bacteria.

## 2. Materials and methods

### 2.1. Bacterial strains and plasmids

*B. anthracis* and *Escherichia coli* strains, plasmid vectors and recombinant plasmids used in this study are listed in Table 1. Construction of the in-frame mutant *B. anthracis* strains used in this study is originally described in Plaut et al. (2014).

**Table 1:** *E. coli*, *B. anthracis* strains and plasmids used in this study.

Strain	Description	Source/reference
***E. coli***		
Arctic Express (DE3) (230192)	*E. coli* B F^−^ *ompT hsdS* (r_B_^−^ m_B_^−^) *dcm*^+^ Tet^r^ *gal* λ(DE3) *endA* Hte [*cpn10 cpn60* Gent^r^]	Agilent
C2925H	*ara-14 leuB6 fhuA31 lacY1 tsx78 glnV44 galK2 galT22 mcrA dcm-6 hisG4 rfbD1 R(zgb210::Tn10)* Tet^S^ *endA1 rspL136* (Str^R^) *dam13::Tn9* (Cam^R^) *xylA-5 mtl-1 thi-1 mcrB1 hsdR2*	NEB
TOP10 (C404010)	F- *mcrA* Δ(*mrr-hsd*RMS-*mcr*BC) Φ80*lac*ZΔM15 Δ *lac*X74 *rec*A1 *ara*D139 Δ(*araleu*)7697 *gal*U *gal*K *rps*L (StrR) *end*A1 *nup*G	Thermo Fisher
***B. anthracis***		
7702	Sterne/pXO1+/pXO2−	Plaut et al. (2014)
BA749	7702 *∆BAS0566*	Plaut et al. (2014)
BA750	7702 *∆sap*	Plaut et al. (2014)
BA751	7702 ∆*eag*	Plaut et al. (2014)
BA752	7702 *∆BAS1792*	Plaut et al. (2014)
BA754	7702 *∆spo0A*	Plaut et al. (2014)
BA755	7702 *∆spo0F*	Plaut et al. (2014)
BAP350	7702 *∆csaB*	Plaut et al. (2014)
DP-B-5747	JB220 *∆spoB*	Plaut et al. (2014)
**Plasmids**		
pSW4	*E. coli*-*B. anthracis* shuttle vector	Pomerantsev et al. (2003)
pSW4-*csaB*	*pSW4::csaB*	This study
pSW4-*spo0A*	*pSW4::spo0A*	This study
pSW4-*spo0B*	*pSW4::spo0B*	This study
pSW4-*spo0F*	*pSW4::spo0F*	This study
pSW4-*sap*	*pSW4::sap*	This study

### 2.2. Culturing of *Bacillus anthracis* strains and heat inactivation

Glycerol stocks of bacteria were used to inoculate 50 mL of tryptic soy broth (TSB). The cultures were grown overnight at 37°C in 250 mL flasks at 150 rotations per minute (rpm). In experiments using heat-killed cells, a 1 mL aliquot was taken from these cultures, diluted to an optical density (OD_600_) of 1.0 and incubated at 98°C for 30 min. Serial dilutions of cells were plated on tryptic soy agar (TSA) to verify non-viability.

### 2.3. Spore preparation

Spores were produced as described in Braun et al. (2020). Briefly, a colony from a fresh overnight TSA plate was inoculated in 50 mL of sporulation medium containing 0.8% nutrient broth supplemented with 0.05 mM MnCl_2_, 0.7 mM CaCl_2_, and 1 mM MgCl_2_ in a 250 mL flask. After cultivation for 72 h at 37°C with shaking at 150 rpm, 3% Tween-80 was added to each flask and incubated for an additional 24 h under the same conditions. Samples were harvested in a 50 mL conical tube by centrifugation at 2000 × g for 10 min. The supernatant was discarded and the pellet was washed twice with 3% Tween-80. Samples were resuspended in 25 mL of 3% Tween-80 and incubated at 37°C for 24 h with shaking at 150 rpm. Phase contrast microscopy was used to approximate the percentage of spores versus vegetative cells in each suspension. When the spore percentage reached >95%, they were harvested by centrifugation at 2000 × g for 10 min, the supernatant was discarded, and the pellet was resuspended in 3 mL of ice-cold ultrapure water and stored at 4°C. The final purity of a given spore preparation was determined by (i) documenting the size and light refractivity by microscopy and (ii) measuring viability following the heating process described above.

### 2.4. DNA isolation

Isolation of DNA from bacterial cultures was performed using the GeneJET Plasmid Miniprep kit (ThermoFisher Scientific) K0502 or the Nanobind High Molecular Weight Extraction kit (Circulomics 102-762-700) according to manufacturer’s recommendations. Extracted nucleic acids were quantified using either the Qubit dsDNA HS Assay (ThermoFisher Scientific Q32851) or the High Sensitivity DNA ScreenTape (Agilent 5,067–5,584).

### 2.5. Whole genome sequencing and variation detection

Whole genome sequencing on the Oxford Nanopore and Illumina platforms and downstream assembly and alignment, were performed as described in Sholes et al. (2023). Single nucleotide polymorphisms (SNPs) and small insertions or deletions were called and filtered as described in Forde et al. (2022). In brief, VarScan v2.4.4 was used with a minimum read depth of 4x, a minimum base quality of 20, and a variant allele frequency ≥ 0.95 (Koboldt et al., 2012). Large structural variations were inspected using sniffles v2.0 (Sedlazeck et al., 2018). All variants were visually validated using IGV (Thorvaldsdóttir et al., 2013).

### 2.6. Cloning of *rbp-gfp* gene fusions

The pASG-105-TST-eGFP-RBP plasmids expressing the receptor binding proteins of phages Wip1 and AP50C and the soluble domain of prophage λBa03 described in Braun et al. (2020) were used as the basis for construction of the new expression constructs used herein. The sequences for the RBPs and corresponding chaperone proteins (when applicable) and for eGFP were provided kindly by Gregor Grass. Insert sequence synthesis, cloning, and sequence validation were performed by Azenta (Burlington, MA). Briefly, an oligonucleotide encoding a start codon and N-terminal HISTAG-thrombin site-Twin-Strep tag was synthesized upstream of the eGFP sequence. A short -CTCGAG- linker sequence was added, followed immediately by the phage RBP sequence. For those RBPs requiring the co-expression of a second protein (RBP_AP50c_ and RBP_Wip1_), the linker sequence -AAGGAGGGAACTAT- was added, followed by the full coding sequence of respective gene. These inserts were cloned into plasmid pET22 using *Nde*I and *Hind*III restriction sites.

### 2.7. Expression and purification of RBP fusion proteins

Protein expression and purification were performed by Olympic Protein Technologies (Seattle, WA). The pET22-eGFP-RBP expression vectors were used to transform (i) *E. coli* Arctic Express DE3 cells (Agilent 230,192) for protein production and (ii) *E. coli* TOP10 (Thermo Fisher C404010) cells for plasmid stock production. Cultures were grown on LB-carbenicillin (100 μg/mL) plates at 37°C overnight prior to single colony selection, outgrowth in liquid culture, and sequence verification using the pET22 entry primers T7 (TAATACGACTCACTATAGGG) and T7 term (GCTAGTTATTGCTCAGCGG).

For protein production, colonies were grown in 25 mL Terrific Broth (TB) with 20 μg/mL gentamicin overnight at 30°C (250 rpm). One liter of autoinduction medium (MagicMedia™ Thermo K6803) was inoculated with 20 mL of the overnight culture and grown at 30°C for 4 hrs before the temperature of the shaken cultures was shifted to 12°C and allowed to proceed overnight. To test for expression, cell pellets were harvested by centrifugation at 10,000 rpm for 10 min at 4°C and lysed using an equal volume of BugbusterHT+ (Millipore Sigma 70,922) and fractionated into soluble and insoluble material. Insoluble fractions were taken up in equal volume as the original culture. All samples were run on a 4–20% Tris-glycine reducing SDS-PAGE gel to ascertain the presence of the tagged protein in the soluble fraction.

For purification, separate cell pellets were resuspended in IMAC breaking buffer (25 mM Tris–HCl, pH 7.9, 500 mM NaCl, 2 mM imidazole, with a complete protease inhibitor tablet and benzonase). The suspensions were lysed via three passages through a microfluidizer. Lysates were centrifuged as above, and, the cleared supernatants were passed through a 0.45 μm filter, and frozen at −80°C.

Thawed lysates were loaded on to a 5 mL Nickel Excel IMAC column (Cytiva) previously equilibrated with IMAC running buffer (50 mM Tris–HCl, pH 8.0, 300 mM NaCl, 2 mM imidazole) using an AKTA instrument. The immobilized proteins were then washed with IMAC running buffer followed by running buffer containing 12 mM imidazole. Once baseline Abs_280_ readings were reached, the bound proteins were eluted using IMAC elution buffer (50 mM Tris–HCl, pH 8.0, 300 mM NaCl, 250 mM imidazole), and peak protein fractions were collected and pooled by Abs_280_ measurements.

Pooled IMAC fractions were adjusted to 10 mM CaCl_2_, and thrombin (Sigma 69,671) was added to a final concentration of 0.24 U/mg of protein. Thrombin reactions were carried out at room temperature for 3 h under conditions of slow rotation. The reaction products were loaded at 3 mL/min on to a 5 mL Strep-Tactin XL Superflow high-capacity column (IBA) previously equilibrated with buffer W (100 mM Tris, pH 8.0, 150 mM NaCl, 1 mM EDTA). Bound samples were washed with buffer W until Abs_280_ measurements reached a steady baseline value. Proteins were eluted using buffer BXT (100 mM Tris, pH 8.0, 150 mM NaCl, 1 mM EDTA, 50 mM biotin). As above, protein peaks were measured by Abs_280_ values and pooled. Overall sample purities were quantified by running a 15 μL aliquot run on an HPLC-SEC column (Tosoh G3000SW) in SEC running buffer (1X PBS) at 200 μL/min. Eluents were run on 4–20% Tris-glycine SDS-PAGE gels (ThermoFisher).

### 2.8. Fluorescence microscopy experiments to assess RBP binding to bacterial cells

A 100 μL aliquot of living or heat-killed vegetative cells (OD_600_ = 1) was added to a 1.5 mL microcentrifuge tube and centrifuged at 5000 × g for 2 min. For spore preparations, 2 × 10^6^ spores were added to 10 mL of Ringer-HEPES buffer (50 mM HEPES, 1.5 mM CaCl_2_, 1.5 mM KCl, 100 mM NaCl, 0.6 mM NaHCO_3_, pH 7.4) and centrifuged at 5000 × g for 2 min. After the supernatant was removed, the pellet was resuspended in 1 mL of Ringer-HEPES buffer then transferred to a 1.5 mL microcentrifuge tube and centrifuged again under the same conditions. The supernatant was removed and the pellet was resuspended in 100 μL of Ringer-HEPES buffer and 5 μg of purified RBP fusion protein was added to the sample. Samples were incubated at room temperature for 5 min then centrifuged and resuspended in 100 μL of Ringer-HEPES buffer. A 3 μL aliquot of the sample was added to a glass microscope slide and imaged by both brightfield and FITC filter. All fluorescence images of samples containing the same phage protein were processed in the same way, with contrast, brightness, and exposure times kept constant. All microscopy images in this manuscript were taken at 100X under oil immersion.

### 2.9. Construction of genetic complementation plasmids

The starting plasmid for the cloning of *B. anthracis* genes was the *E. coli*-*B. anthracis* shuttle vector, pSW4, the construction of which was previously described (Pomerantsev et al., 2003). The coding sequences of the bacterial genes of interest were individually synthesized, inserted into pSW4 linearized using *Bgl*II and *Blp*I, and sequence verified by Azenta Life Sciences (Burlington, MA). Constructs were then transformed into competent *dam^−^*/*dcm^−^ E. coli* cells (NEB C2925H) and purified to yield unmethylated plasmid stocks.

### 2.10. Electroporation of plasmids into *Bacillus anthracis* strains

To make electrocompetent cells, *B. anthracis* wild type or mutant strains were grown overnight on LB plates containing 1% w/v glucose (LBG agar). Colonies were inoculated into 1 mL of LBG medium and incubated for 10 min at 37°C and 225 rpm before being spiked into a larger 25 mL LBG culture. The culture was incubated and shaken until the OD_600_ reached 0.15–0.25. Cells were centrifuged at 4000 × g for 5 min and washed three times with electroporation buffer (10% sucrose, 15% glycerol, 2 mM potassium phosphate buffer, pH 8.0–8.4). Cells were resuspended in 200 μL of electroporation buffer and transferred to a 0.2 cm electroporation cuvette. After incubation on ice for 10 min, approximately 0.5 μg of unmethylated plasmid was added into the cuvette and mixed gently with the cells. Cells were pulsed once at 1.77 kV with a mean time constant between 3.5–5 msec, then 1 mL of S.O.C medium was added to the cells. Cells were transferred to a FALCON 14 mL tube and incubated at 37°C for 1 h. A 200 μL aliquot was plated on LBG-kanamycin plates and incubated for 16 h at 37°C.

## 3. Results

### 3.1. Whole genome sequencing of *Bacillus anthracis* Sterne mutants verifies gene deletions

**Figure 1:**
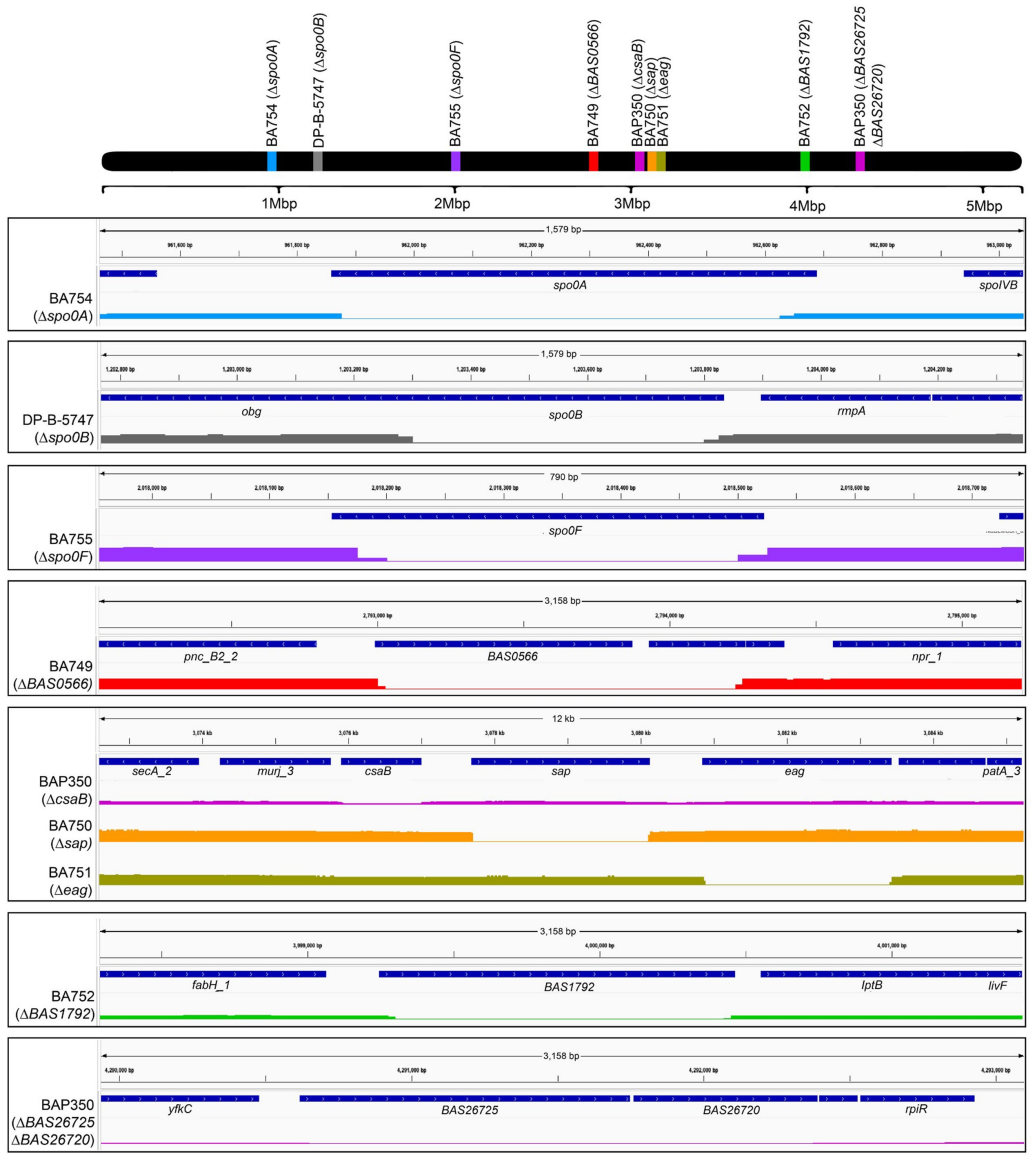
Genomic maps of wild type and mutant strains of *B. anthracis* Sterne 7702 indicating the presence of deletions in different locations. The top black bar represents the bacterial chromosome with the relative locations of the genes targeted for deletion in the mutants. The bottom blocks zoom in on the individual regions of each deletion with genome positions provided on top, relevant open reading frames in the middle, and sequence coverage of respective locus with flanking regions on the bottom. The mutant bars are color coded to match with the genes indicated on the whole chromosome map on top. Black boxes are to aid in visual differentiation of different strains. The secondary, off-target hitch-hiker deletions in the BAP350 strain (ORFs *BAS26725* and *BAS26720*) are indicated in the bottom-most box.

A transposon insertion screen previously identified several genes in *B. anthracis* Sterne 7702 that appeared to be necessary for AP50c phage adsorption (Plaut et al., 2014). These included *sap*, *spo0A*, and *spo0F* (discussed in the Introduction section), as well genes encoding the transcriptional regulator BAS0566 and the branched amino-acid ABC transporter BAS1792. Each of these genes, as well as two genes in the same operon as *sap*, (*csaB* and *eag*, the latter of which is an alternate S-layer protein), were then individually deleted using markerless allelic exchange, and a final mutant strain was created that lacked the additional sporulation gene *spo0B* (Plaut et al., 2014). Recently, the full sequences of the genomes of these strains were published (Sholes et al., 2023). We found that each of these mutant strains contained the expected deletions of the targeted genes (Figure 1), in addition to individual SNPs or deletions present elsewhere in the genome (Supplementary Table S1). The BAP350 mutant (Δ*csaB*) also contained two additional deletions in genes *BAS2672*5 and *BAS26720* (Figure 1, bottom block). All these “hitch-hiker” mutations were taken into consideration when examining the results of RBP binding experiments described below.

### 3.2. Expression and purification of phage receptor binding proteins

**Figure 2:**
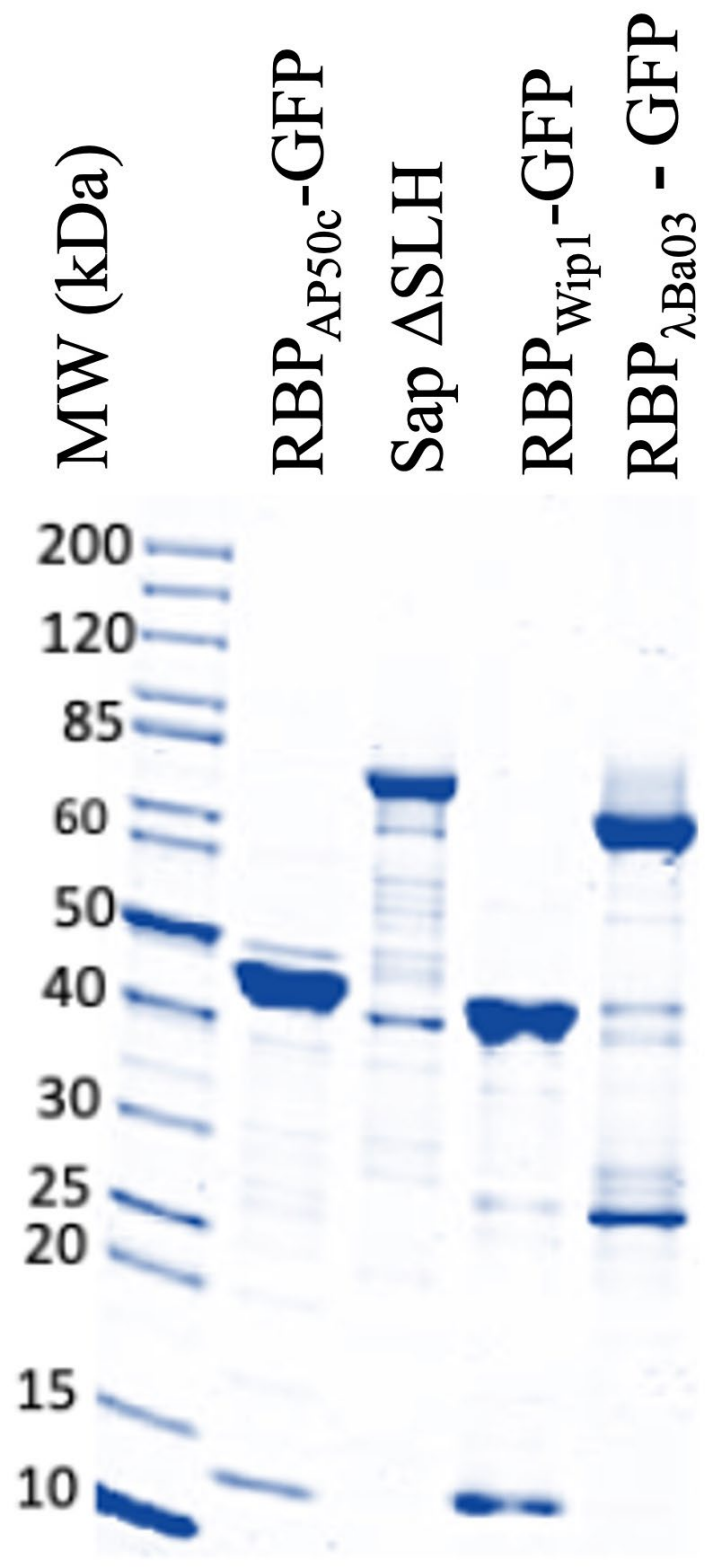
Production and purification of the three GFP-tagged phage receptor binding proteins and the untagged *sap*∆SLH domain (not used in current study). Five microgram aliquots of the final preparations of each purified product were run on a denaturing 4–20% Tris-glycine gel and imaged using a LI-COR instrument (LI-COR Biosciences). The <15 kDa bands in the RBP_AP50c_-GFP (P28) and RBP_Wip1_-GFP (P23) lanes represent the co-expressed chaperone proteins P29 and P24, respectively.

Earlier work by Braun et al. described the identification, cloning, expression, and purification of the putative receptor binding proteins (RBPs) of phages Wip1 (P23), AP50c (P28), γ (Gp14) and a soluble domain of λBa03 (BA4079 Δ1-120) (Braun et al., 2020). Here, we expanded on this work and developed a plasmid system in which each RBP could be expressed with GFP fused to the N-terminus. These constructs additionally contained a His-thrombin site and *Twin-Strep-tag* epitope to aid in purification. For the plasmids expressing the Wip1 or AP50c RBP-GFP fusion proteins, a second protein encoded immediately downstream in the phage genome was co-expressed based on previous results suggesting these secondary proteins were required for RBP function (Braun et al., 2020). Proteins were produced in Arctic Express *E. coli* cells and following autoinduction, lysates were separated into soluble and insoluble fractions. As all proteins of interest were soluble, these samples were fractionated using immobilized nickel affinity chromatography columns. The purity of each fraction was measured by Abs_280_ and size exclusion chromatography. Final preparations were visualized on a 4–20% Tris-glycine gel (Figure 2). These proteins are henceforth referred to as RBP_Wip1_-GFP, RBP_AP50c_-GFP, and RBP_λ03Δ1-120_-GFP.

### 3.3. Binding of phage RBPs to *Bacillus anthracis* mutants

**Figure 3:**
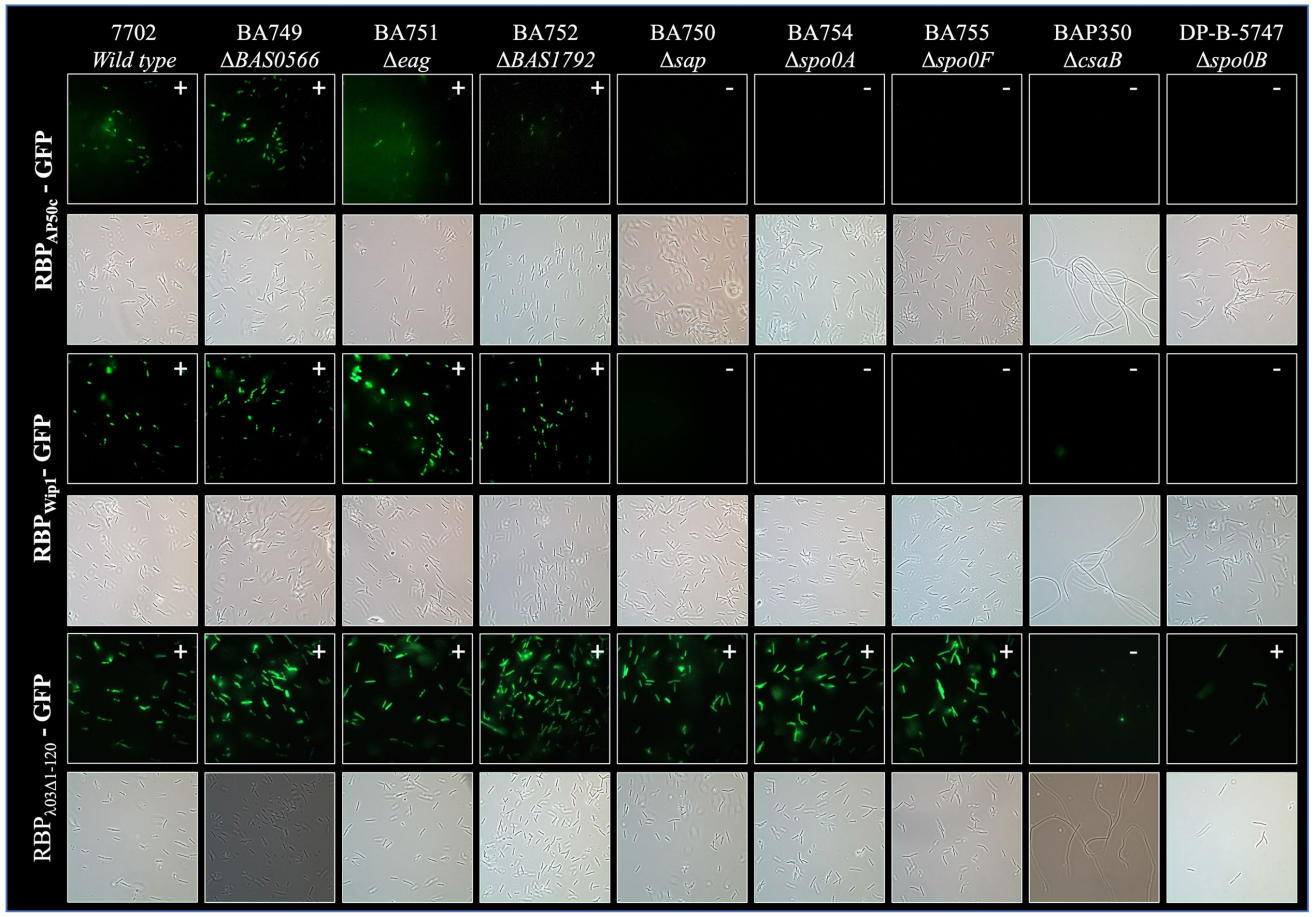
Representative fluorescence and brightfield images showing binding of RBP_AP50c_-GFP, RBP_Wip1_-GFP, and RBP_λ03Δ1-120_-GFP to cultures of vegetative *B. anthracis* Sterne mutants. All slides were prepared using cultures harvested at OD_600_ = 1.0 following a five-minute incubation with 5 μg of purified phage proteins. Note the filamentous morphology of the BAP350 strain lacking *csaB*.

The protein preparations containing the recombinant, tagged phage RBPs were next tested for binding against the panel of sequence-verified mutant strains described in Figure 1. As previous work suggested that phage RBP binding (i) is dependent on the growth phase of the bacteria and (ii) is strong for RBP_Wip1_, RBP_AP50c_, and RBP_λ03Δ1-120_ during logarithmic growth (Braun et al., 2020), all experiments were performed using cells harvested during this period. Cells were not otherwise synchronized. As expected, all three phage RBPs bound to wild type Sterne strain 7702, as well as to mutants lacking expression of *BAS0566* (BA749), *eag* (BA751), and *BAS1792* (BA752) (Figure 3). In support of earlier work with whole phage AP50c (Plaut et al., 2014), the deletion of *sap*, *csaB*, or any one of the three sporulation genes *spo0A*, *spo0B*, or *spo0F* also resulted in the loss of RBP_AP50c_-GFP binding. The same binding profile was observed with the RBP_Wip1_-GFP. Interestingly, the putative RBP_λ03Δ1-120_-GFP bound to all mutant strains with the exception of BAP350, which lacks *csaB*.

We next looked to determine whether the binding of the phage RBPs would be affected by heating of the bacterial culture. Aliquots of mid-log cultures were heated for 30 min at 98°C and plated to verify inactivation prior to incubation with each tagged phage RBP. This process resulted in the loss of RBP_AP50c_-GFP and RBP_Wip1_-GFP binding to all strains (Supplementary Figure S1). The binding of RBP_λ03Δ1-120_-GFP to bacterial cells was not affected by heating, with the exception of the *csaB* mutant.

As *B. anthracis* cells show significant changes in surface protein expression following sporulation when compared to vegetative cells (Chateau et al., 2020), we were interested to determine whether the bacterial receptors for the RBPs were present on spore surfaces. To this end, tagged RBPs were incubated with spores prepared from each mutant strain. Of those strains that successfully sporulated, no phage RBP binding was observed (Supplementary Figure S2). A summary of the binding profiles for each RBP to each mutant strain under all three conditions can be seen in Table 2.

**Table 2:** Binding phenotypes observed for each of the *B. anthracis* Sterne mutant cultures when incubated with RBPs-GFP fusion proteins.

Strain	Phenotype	Live vegetative cells			Heat-killed vegetative cells			Spores		
		RBP_AP50c_	RBP_Wip1_	RBP_λ03Δ1–120_	RBP_AP50c_	RBP_Wip1_	RBP_λ03Δ1–120_	RBP_AP50c_	RBP_Wip1_	RBP_λ03Δ1–120_
7702	Wild-Type	+	+	+	−	−	+	−	−	−
BA749	*ΔBAS0566*	+	+	+	−	−	+	−	−	−
BA751	*Δeag*	+	+	+	−	−	+	−	−	−
BA752	*ΔBAS1792*	+	+	+	−	−	+	−	−	−
BA750	*Δsap*	−	−	+	−	−	+	−	−	−
BA754	*Δspo0A*	−	−	+	−	−	+	N/A	N/A	N/A
BA755	*Δspo0F*	−	−	+	−	−	+	N/A	N/A	N/A
BAP350	*ΔcsaB*	−	−	−	−	−	−	−	−	−
DP-B-5747	*Δspo0B*	−	−	+	−	−	+	N/A	N/A	N/A

### 3.4. Expression of *csaB* in *trans* restores wild-type phage RBP binding profiles

**Figure 4:**
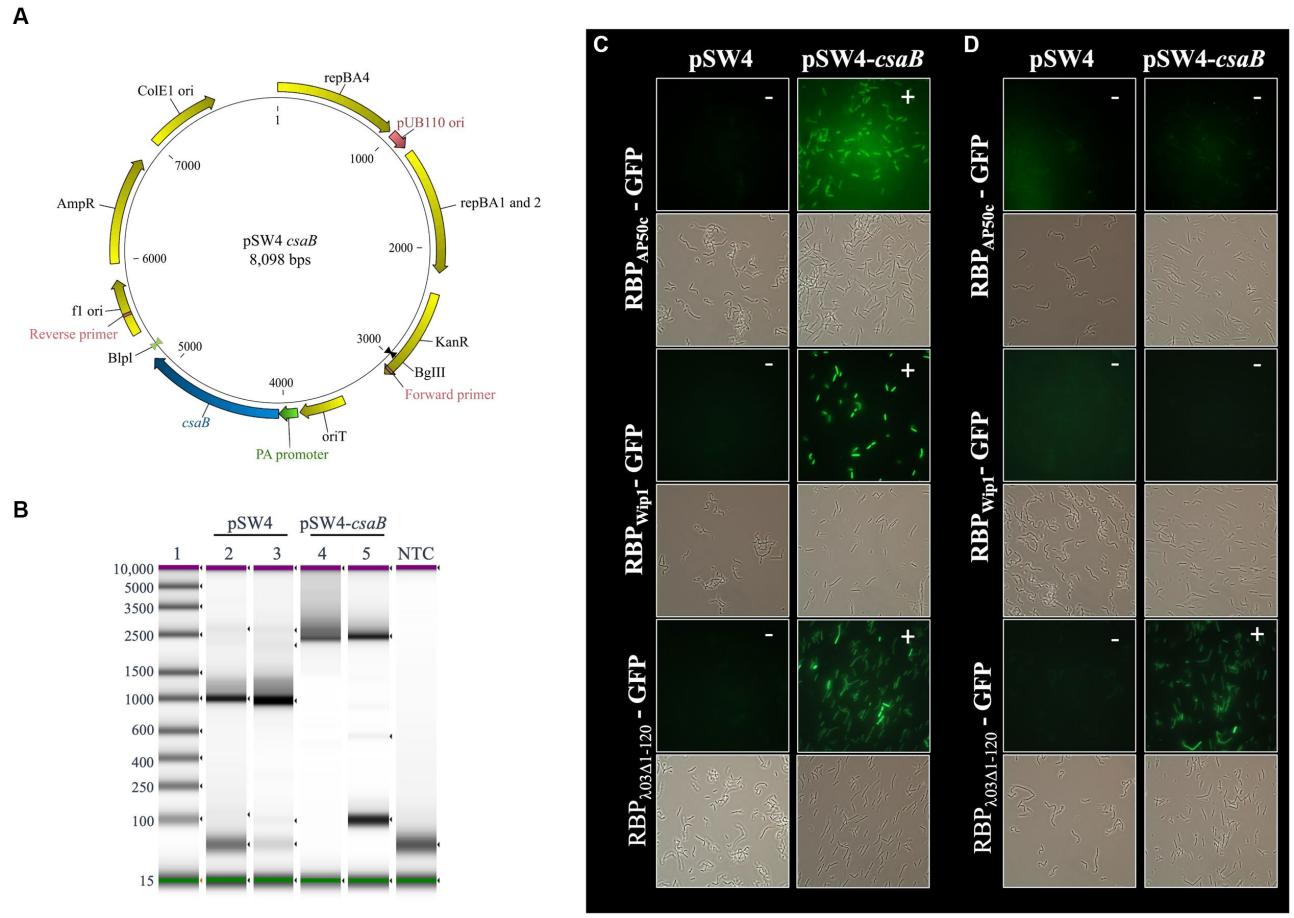
Expression of *csaB* in *trans* rescues (i) RBP_AP50c_-GFP and RBP_Wip1_-GFP binding to live cultures of ∆*csaB* mutant strain BAP350 and (ii) RBP_λ03Δ1-120_-GFP binding to heat-killed cultures. **(A)** Plasmid map for pSW4-*csaB*. **(B)** PCR amplicon products show the presence of the expression vector/complementation plasmid in strain BAP350. Lane 1: base pair ladder. Lanes 2 and 4: Stock plasmid preparations. Lane 3 and 5: Plasmids isolated from electroporated BAP350 cultures. NTC indicates no template control. Representative fluorescent microcopy images show binding patterns of tagged phage RBPs to **(C)** vegetative, or **(D)** heat-killed preparations of BAP350 with and without *csaB* complementation. Small white notations in upper right-hand corner of each image indicate the presence (+) or absence (−) of binding.

The only mutant strain that resulted in the loss of binding for all three phage RBPs with live vegetative cells was BAP350 (∆*csaB*). To ensure that this phenotype was due only to this mutation and not to the two hitch-hiker mutations and/or SNPs present in this strain, we expressed *csaB* in *trans* from a complementation plasmid. The coding sequence for *csaB* was cloned into the pSW4 shuttle expression vector as described in the Materials and Methods (Figure 4A) and introduced into BAP350 (*ΔcsaB*) or the wild type 7702 strain by electroporation. The presence of the plasmid in electroporated cells was verified by PCR (Figure 4B), and binding studies were repeated as described above. Supplying *csaB* protein in *trans* in BAP350 indeed rescued wild-type binding profiles of all three RBPs (Figures 4C,D), suggesting that the phenotype seen with the *csaB* mutant strain in earlier experiments was due to the lack of *csaB* expression alone. It should additionally be noted that expression of *csaB* in *trans* also reversed the mucoid/filamentous morphology phenotype observed in BAP350. It appears that the presence of pSW4 vector by itself, is somewhat partially reverting the mucoid phenotype as observed by the fluffy nature of the culture in the tube (Supplementary Figure S3).

### 3.5. Expression of *sap* and *spo0* genes in *trans* rescues RBP_Wip1_-GFP, RBP_AP50c_-GFP binding

**Figure 5:**
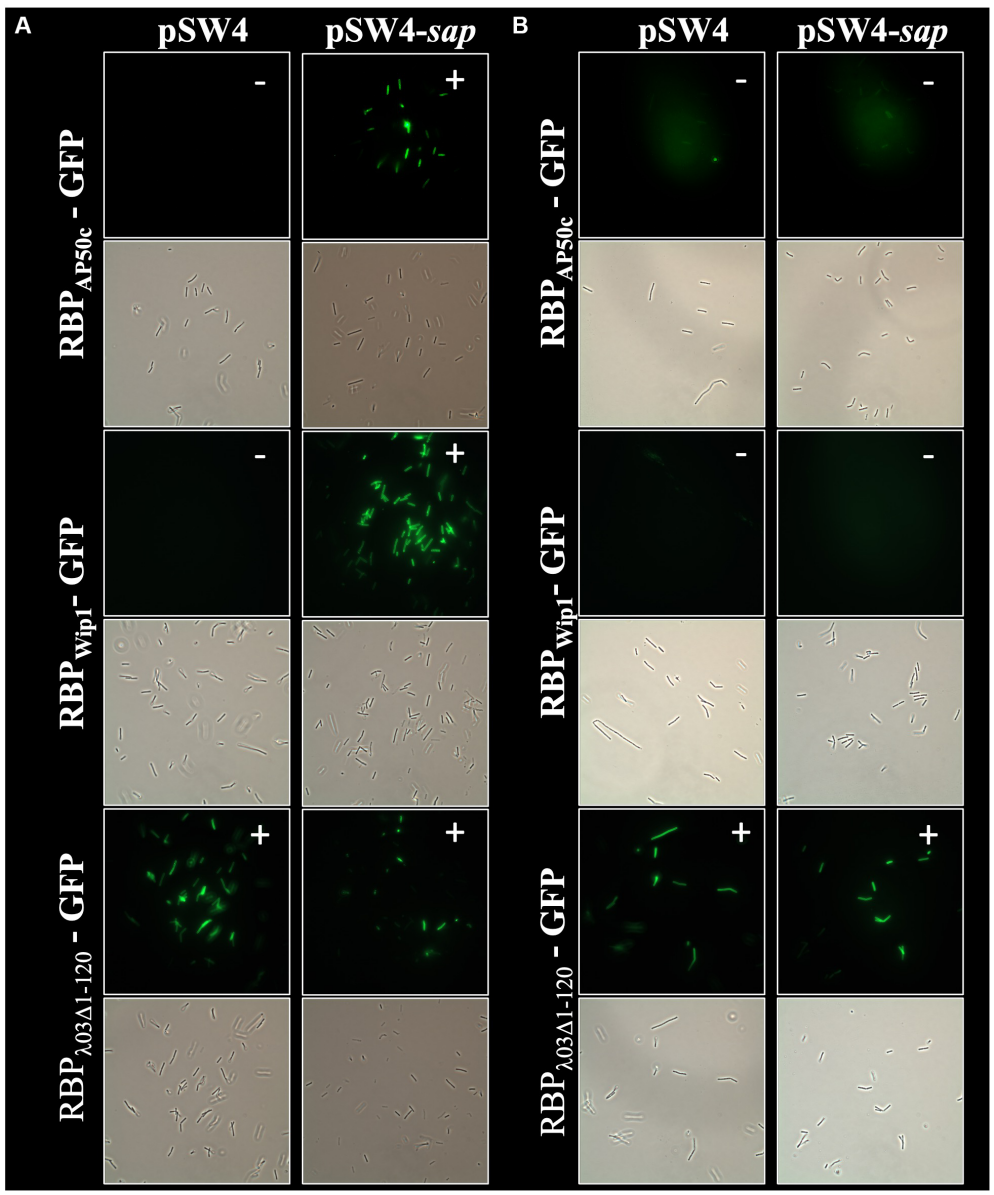
Expression of *sap* in *trans* rescues RBP_AP50c_-GFP and RBP_Wip1_-GFP binding to ∆*sap* mutant strain BA750. Representative fluorescent microcopy images show binding patterns of tagged phage RBPs to **(A)** vegetative, or **(B)** heat-killed preparations of BA750 with and without *sap* complementation. Small white notations in upper right-hand corner of each image indicate the presence (+) or absence (−) of binding.

**Figure 6:**
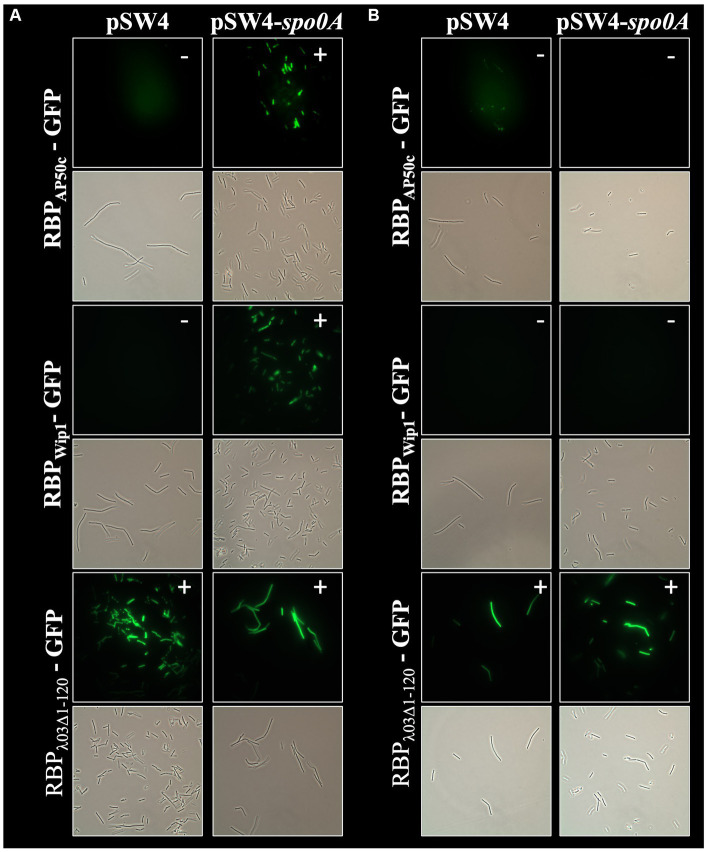
Expression of *spo0A* in *trans* rescues RBP_AP50c_-GFP and RBP_Wip1_-GFP binding to ∆*spo0A* mutant strain BA754. Representative fluorescent microcopy images show binding patterns of tagged phage RBPs to **(A)** vegetative, or **(B)** heat-killed preparations of BA754 with and without *spo0A* complementation. Small white notations in upper right-hand corner of each image indicate the presence (+) or absence (−) of binding.

**Figure 7:**
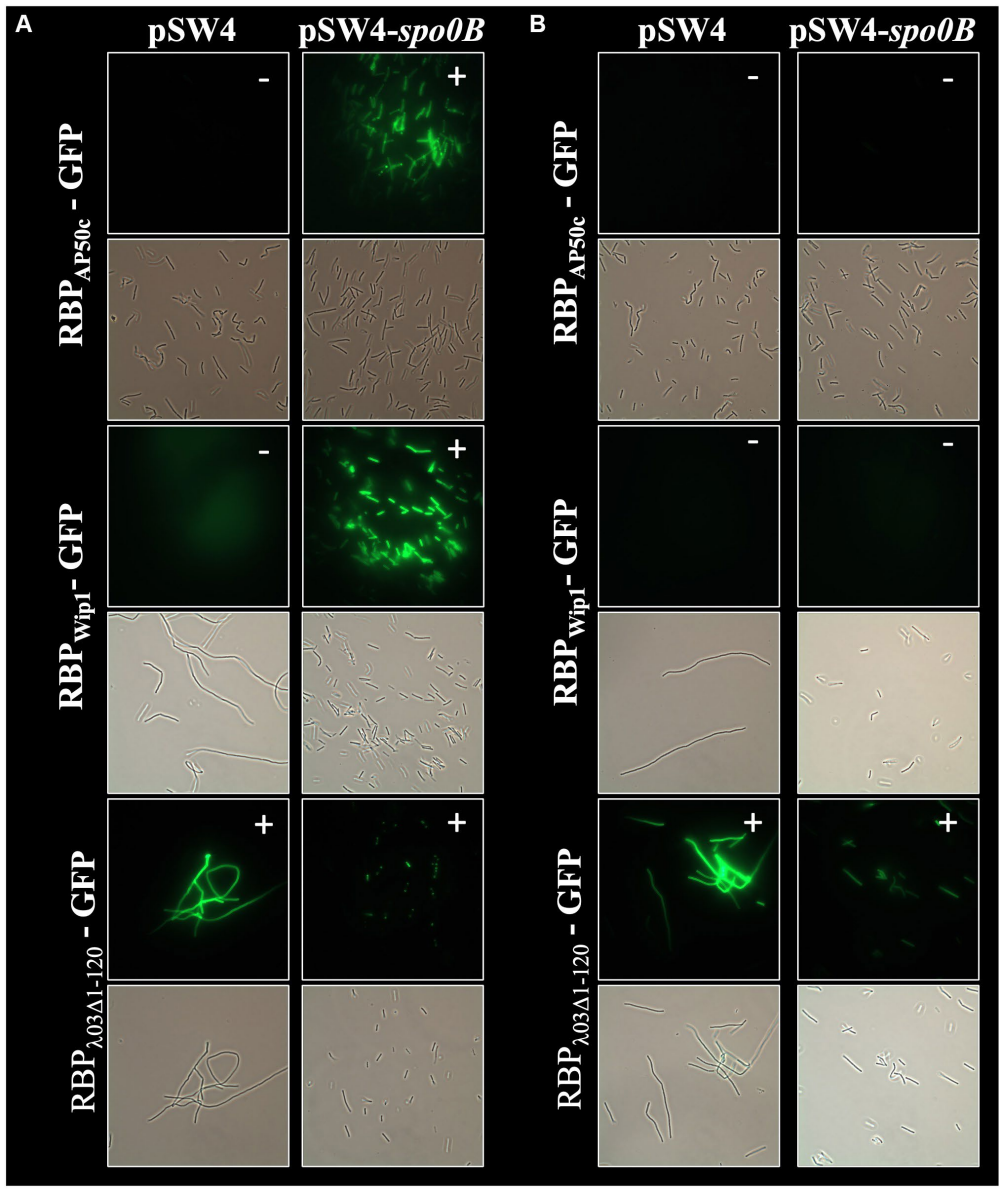
Expression of *spo0B* in *trans* rescues RBP_AP50c_-GFP and RBP_Wip1_-GFP binding to ∆*spo0B* mutant strain DP-B-5747. Representative fluorescent microcopy images show binding patterns of tagged phage RBPs to **(A)** vegetative, or **(B)** heat-killed preparations of DP-B-5747with and without *spo0B* complementation. Small white notations in upper right-hand corner of each image indicate the presence (+) or absence (−) of binding.

**Figure 8:**
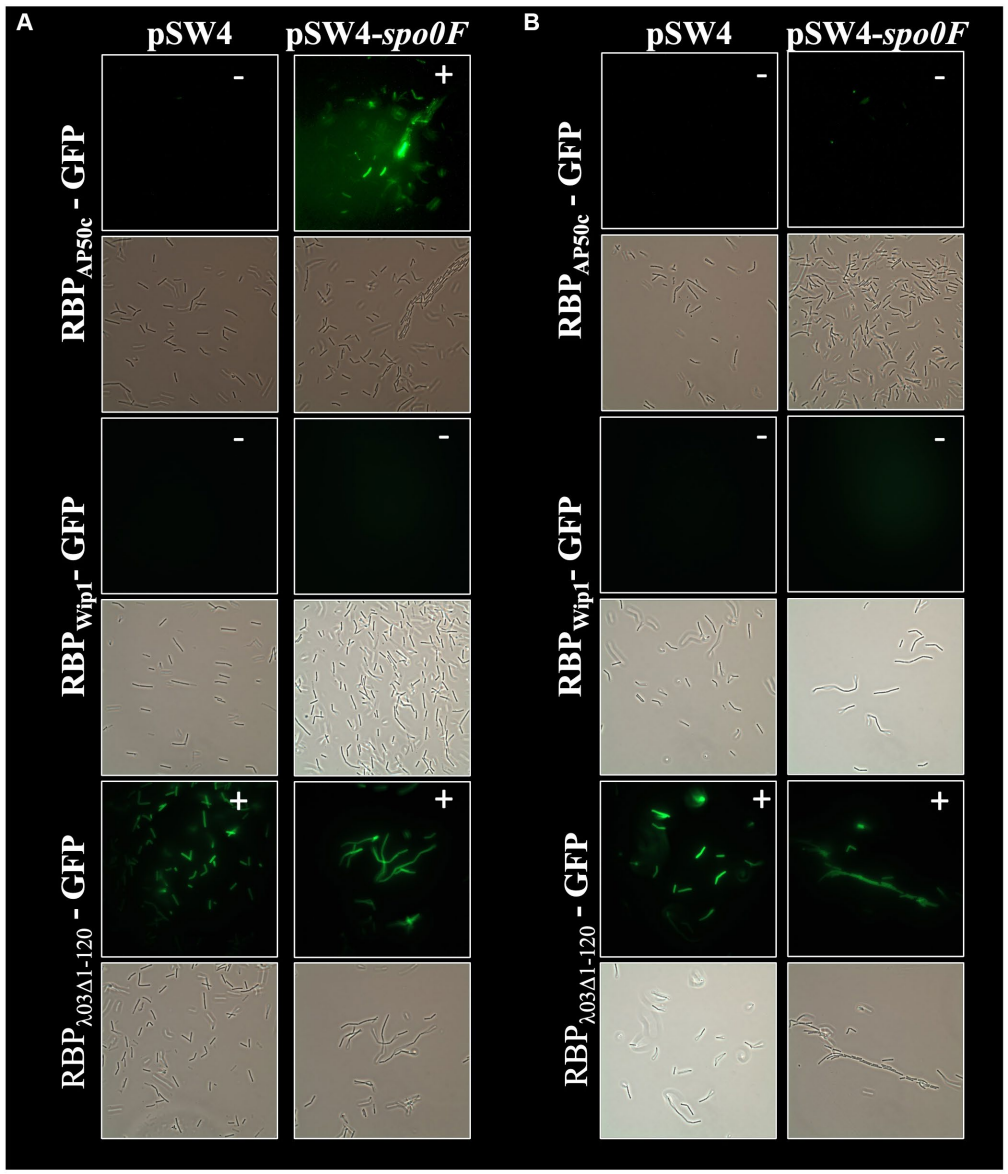
Expression of *spo0F* in *trans* does rescue RBP_AP50c_-GFP binding and does not rescue RBP_Wip1_-GFP binding to ∆*spo0F* mutant strain BA755. Representative fluorescent microcopy images show binding patterns of tagged phage RBPs to **(A)** vegetative, or **(B)** heat-killed preparations of BA755 with and without *spo0F* complementation small white notations in upper right-hand corner of each image indicate the presence (+) or absence (−) of binding.

In addition to the role of *csaB* in the binding of all 3 phage RBPs, the RBP_Wip1_-GFP and RBP_AP50c_-GFP proteins also required the expression of *sap* and the sporulation genes *spo0A*, *spo0B*, and *spo0F* for successful binding (Figure 3). This had been observed previously with whole phage particles (Plaut et al., 2014). A similar set of complementation experiments was carried out for all of these mutant strains, each of which were electroporated with either the empty pSW4 vector or the appropriate complementation construct. The successful electroporation of each plasmid was verified both by PCR (Supplementary Figure S4) and by the restoration of sporulation in mutant strains (Supplementary Figure S5). In the bacteria containing the complementation plasmids expressing *sap* (complementation in strain BA750) (Figure 5), *spo0A* (BA754, Figure 6), and spo0B (DP-B-5747, Figure 7), the wild-type binding profile was restored. In experiments with the *spo0F* mutant (BA755), however, complementation rescued RBP_AP50c_-GFP binding to live vegetative cells but did not rescue the binding of the RBP_Wip1_-GFP (Figure 8). RBP_λ03Δ1-120_-GFP binding was unaffected in these mutants or the corresponding complemented strains.

## 4. Discussion

In this study, we have investigated the genetic requirements for the binding of bacteria and phage in a *B. anthracis* model system. We assessed the binding of three different phage RBPs with a collection of *B. anthracis* mutant strains with the goal of better understanding the underlying biology driving these interactions. While there has been previous work on the binding of these 3 phage RBPs to wild type bacteria (Braun et al., 2020), two questions remained unanswered; namely, (i) is there genetic evidence for these binding pairs? and (ii) is this binding affected by mutations in a specific set of bacterial genes? These questions are critical as they relate directly to the potential for development of phage resistance and the failure of assays that rely on this binding. Here we provide experimental evidence that answers these questions.

Using a set of GFP-tagged recombinant proteins, we recapitulated earlier results derived from whole phage particle experiments. These results indicated that: (i) the RBP_AP50c_ likely binds the bacterial receptor *sap*, (ii) this binding interaction requires the expression of *csaB* and the sporulation genes *spo0A*, *spo0B*, and *spo0F*, and (iii) the binding is ablated following heat inactivation. While the putative RBP_Wip1_-GFP does not share extensive sequence similarity to that of RBP_AP50c_-GFP, it does contain a small region of identical amino acids at its C-terminal, and there does exist a striking similarity in the product of genes immediately downstream of the RBPs in both phage genomes (Braun et al., 2020). Our study here suggests that Wip1 likely uses the same bacterial protein, *sap*, as its receptor, and that *csaB* and the same three sporulation genes are necessary for this interaction. The role of *csaB* in this binding interaction is not surprising, as it has a known role in anchoring *sap* to the S-layer (Mesnage et al., 2000). The role of the sporulation genes, however, is less intuitive. We hypothesize that the activity of Spo0A, a major transcriptional regulator (Burbulys et al., 1991), has a role in the expression or stability of *sap* or the S-layer in general. This concept has been introduced in previous work (Plaut et al., 2014), and our results support an expansive role for the sporulation phosphorelay in overall cell state. The successful rescue of wild-type binding profiles, except *spo0F* with RBP_Wip1_-GFP, upon complementation with the protein absent in a given mutant strain suggests that the minor additional “hitch-hiker” mutations play no role in the phenotypes seen in each of these strains. Additionally, we could find no evidence in the literature supporting the potential involvement of these genetic loci in phage binding, S-layer function, or sporulation. The anomaly with the failure of rescue of *spo0F* complementation with RBP_Wip1_-GFP binding is not clear at this time.

Our results showing a lack of binding of RBP_AP50c_-GFP and RBP_Wip1_-GFP to heat-inactivated vegetative cells is somewhat unexpected based on earlier published results (Braun et al., 2020). We hypothesize that this difference could be attributed to strain- and experiment-specific differences. Even in the previously published studies, binding of each RBP to heat-inactivated cells was somewhat diminished compared to wild type cells, and it varied based both on the method of inactivation and the RBP in question (Braun et al., 2020).

Similar to previous works, and unlike the results observed with RBP_AP50c_-GFP and RBP_Wip1_-GFP, we found that prophage RBP_λ03Δ1-120_-GFPbinding was not affected by heat inactivation. However, while earlier work also indicated that *csaB* was *not* involved in the growth of phage γ, which shows high sequence homology to λBa03 (Davison et al., 2005), we find here that the absence of *csaB* ablated RBP_λ03Δ1-120_-GFP binding. The sequence similarity between phage γ and prophage λBa03 suggests that λBa03 shared the same bacterial host receptor as gamma (GamR). Our results, however, suggest that either (i) GamR is not accessible to the RBP_λ03Δ1-120_-GFP in the absence of *csaB* (but it is accessible to the whole γ phage particle). The *csaB* mutants are known to secrete an extracellular flocculent material and in fact, scanning electron microscopic analysis of this mutant revealed the presence of a thin coating of an extracellular material on the outer cell surface (Sozhamannan et al., 2008); it is possible that this layer prevents access of RBP_λ03Δ1-120_-GFP to the bacterial receptor, or (ii) GamR is not the bacterial receptor for λBa03. If the former is true, GamR may be somehow shielded from heat denaturation, while if the latter is true, it may be that the receptor for RBP_λ03Δ1-120_-GFP is not proteinaceous in nature, as most proteins lose secondary structure following high temperature exposure. Recent work has in fact suggested that other γ-like phages utilize sugar moieties as part of their binding process (Nakonieczna et al., 2022) and we postulate that this may be the case for λBa03. Our future studies will examine this possibility, and further probe direct protein–protein interactions between phage RBPs and bacterial receptor proteins.

## Data availability statement

The data presented in the study are deposited in GenBank, accession numbers CP110279, JAOZJJ000000000, CP110281, JAOZJK000000000, JAOZJL000000000, JAOZJM000000000, CP110283, CP110285, and CP110287.

## Author contributions

SF: Conceptualization, Formal analysis, Investigation, Writing – review & editing. ST: Conceptualization, Formal analysis, Investigation, Writing – review & editing. SaS: Conceptualization, Formal analysis, Investigation, Writing – review & editing. SH: Conceptualization, Formal analysis, Investigation, Writing – review & editing. RP: Investigation, Writing – review & editing. KV: Project administration, Writing – review & editing. MW: Investigation, Writing – review & editing. EE: Investigation, Writing – review & editing. BN: Funding acquisition, Writing – review & editing. ShS: Conceptualization, Data curation, Formal analysis, Funding acquisition, Project administration, Writing – original draft, Writing – review & editing. SG: Conceptualization, Formal analysis, Investigation, Writing – original draft, Writing – review & editing.
