# Supplementary material for: Genetic evidence for the interaction between Bacillus anthracis-encoded phage receptors and their cognate phage-encoded receptor binding proteins
Source: Front Microbiol. 2023 Oct 31;14:1278791. doi: 10.3389/fmicb.2023.1278791 (PMC10644760; doi:10.3389/fmicb.2023.1278791)
Supplement: Supplementary file 1 [file Data_Sheet_1.docx]

Supplementary Material


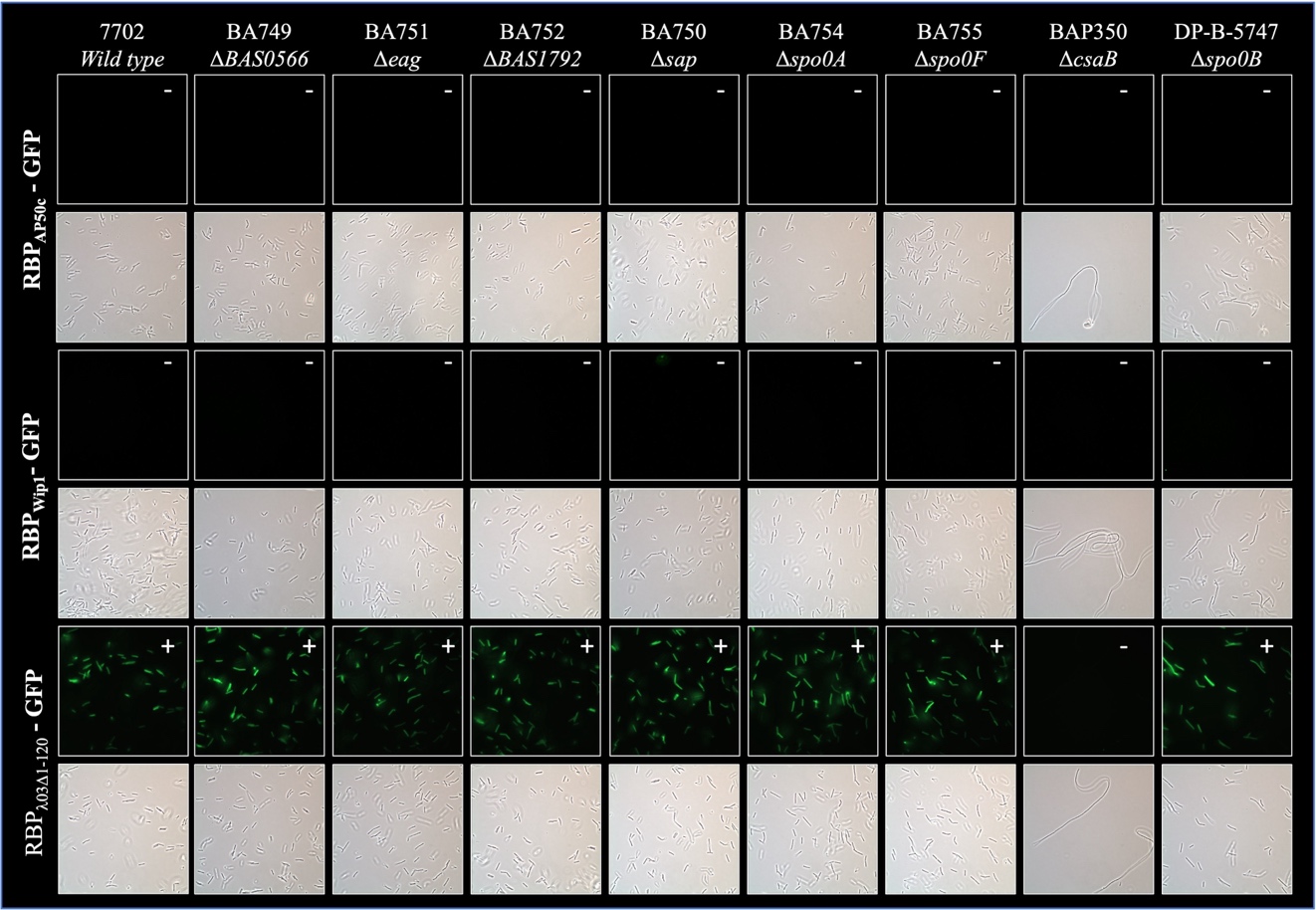


**Supplementary Figure 1:** Representative fluorescence and brightfield images showing binding of RBP_AP50c_-GFP, RBP_Wip1_-GFP, and RBP_λ03Δ1-120_-GFP to cultures of heat-killed vegetative *B. anthracis* Sterne mutants. Small white notations in upper right-hand corner of each image indicate the presence (+) or absence (-) of binding.


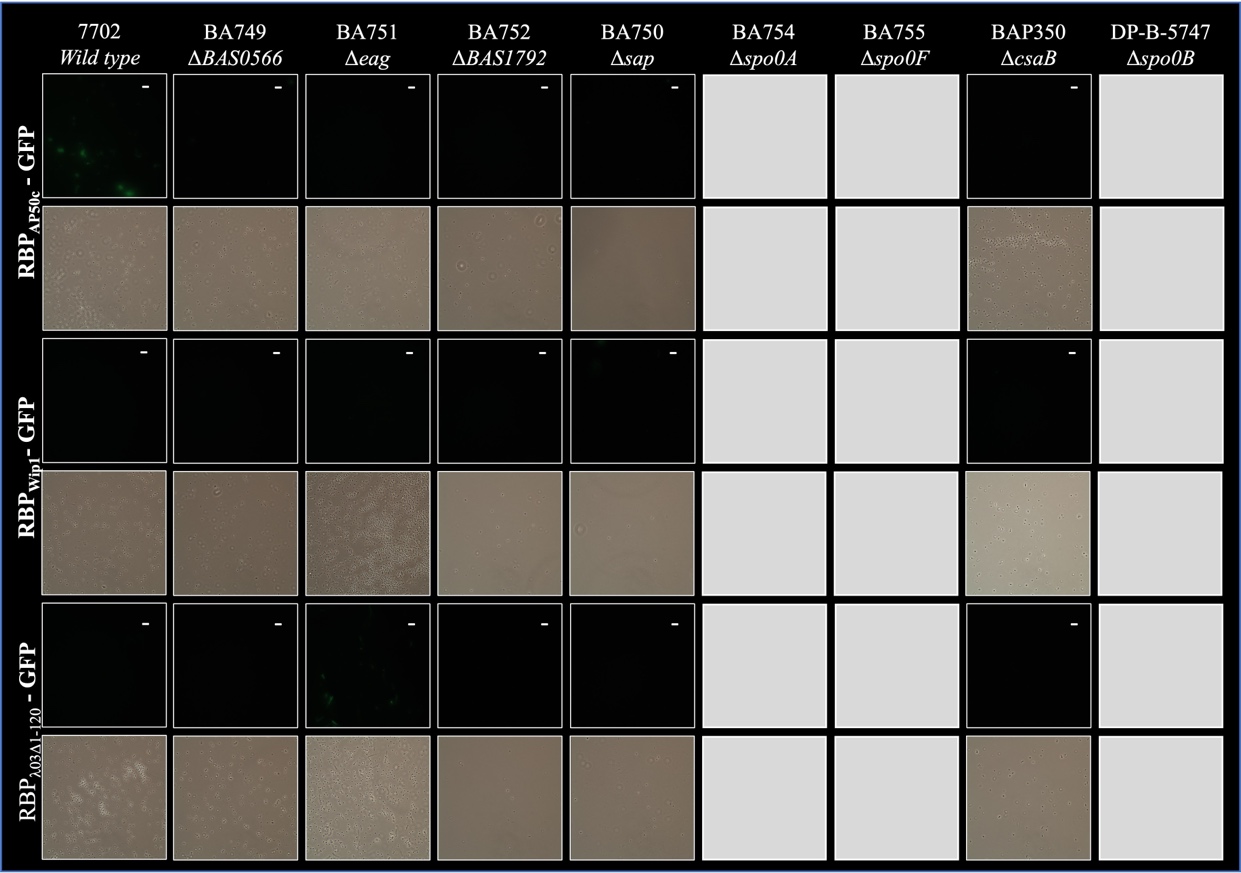


**Supplementary Figure 2.** Representative fluorescence and brightfield images showing binding of RBP_AP50c_-GFP, RBP_Wip1_-GFP, and RBP_λ03Δ1-120_-GFP to spore preparations of *B. anthracis* Sterne mutants. Grey boxes indicate that the strain did not sporulate. Small white notations in upper right-hand corner of each image indicate the (-) of binding.


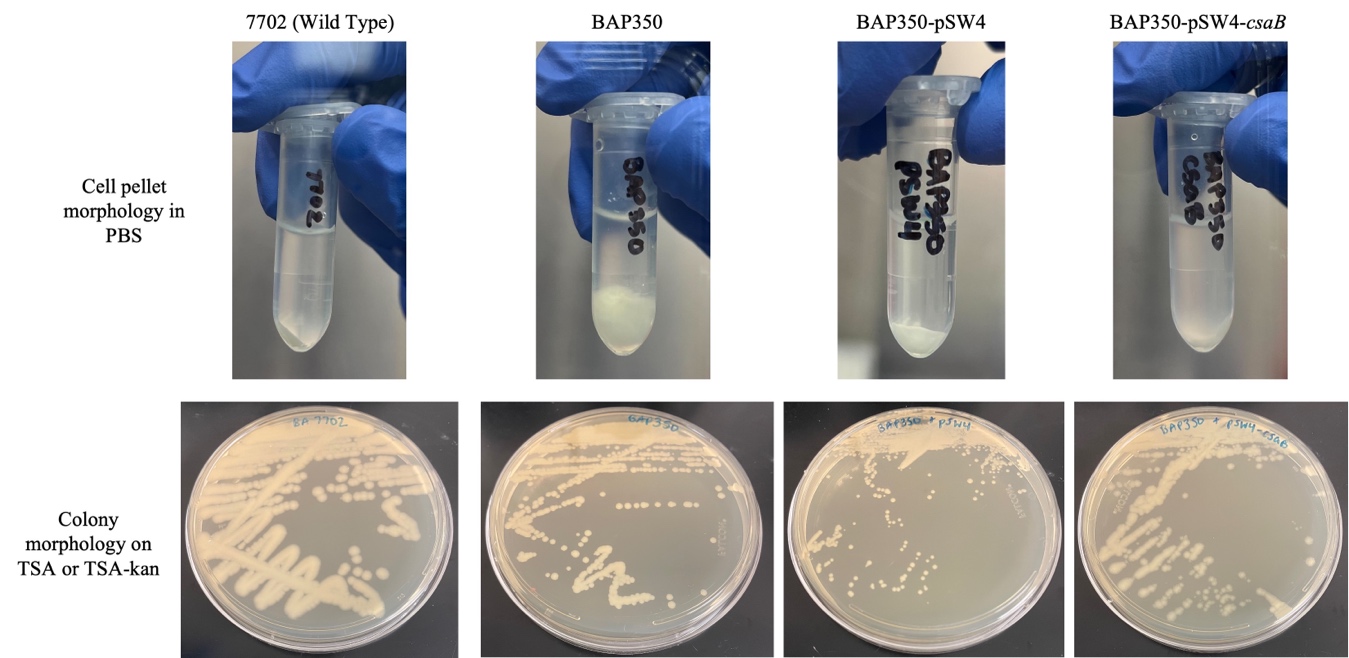


**Supplementary Figure 3.** BAP350 strain shows mucoid and filamentous morphology observable in both cell pellets (top row) and colony streaks (bottom row) which is lost upon electroporation with pSW4-*csaB*. Strains 7702 and BAP350 were plated on TSA. BAP350-pSW4 and BAP35-pSW4-*csaB* were plated on TSA-kanamycin.


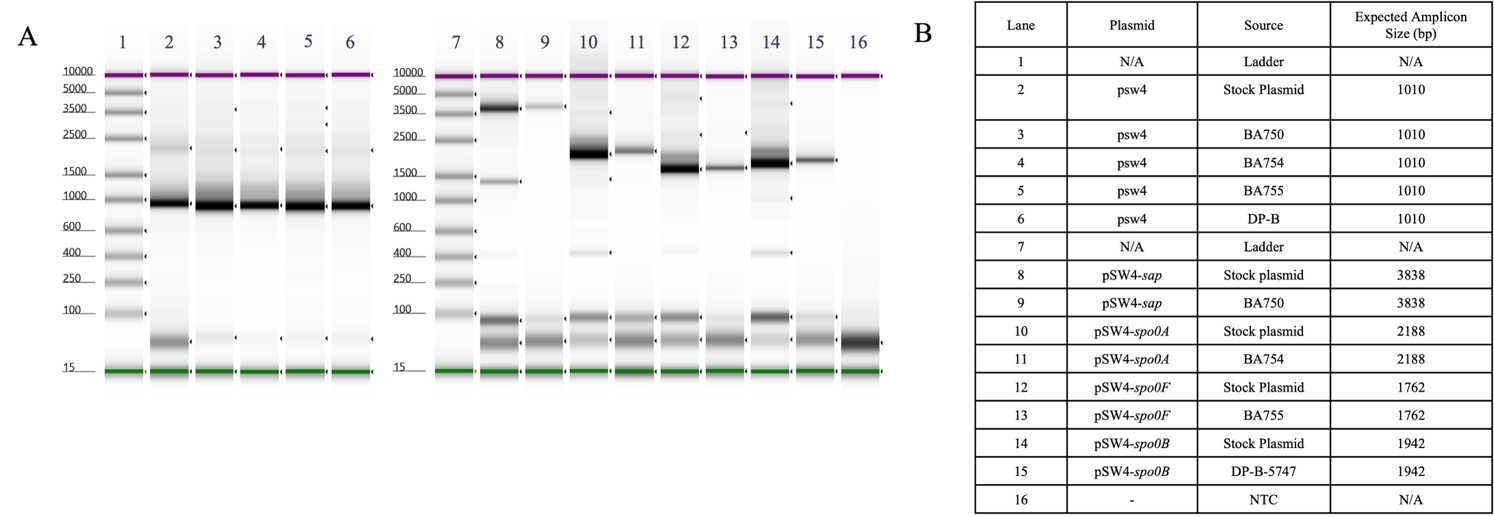


**Supplementary Figure 4:** PCR amplicon products show the successful introduction of expression vectors into corresponding *B. anthracis* mutant strains. (A) PCR amplicon products for each plasmid as prepared from electroporated *B. anthracis* mutant strain. (B) Table summarizing expected size of amplicon products. NTC = no template control.


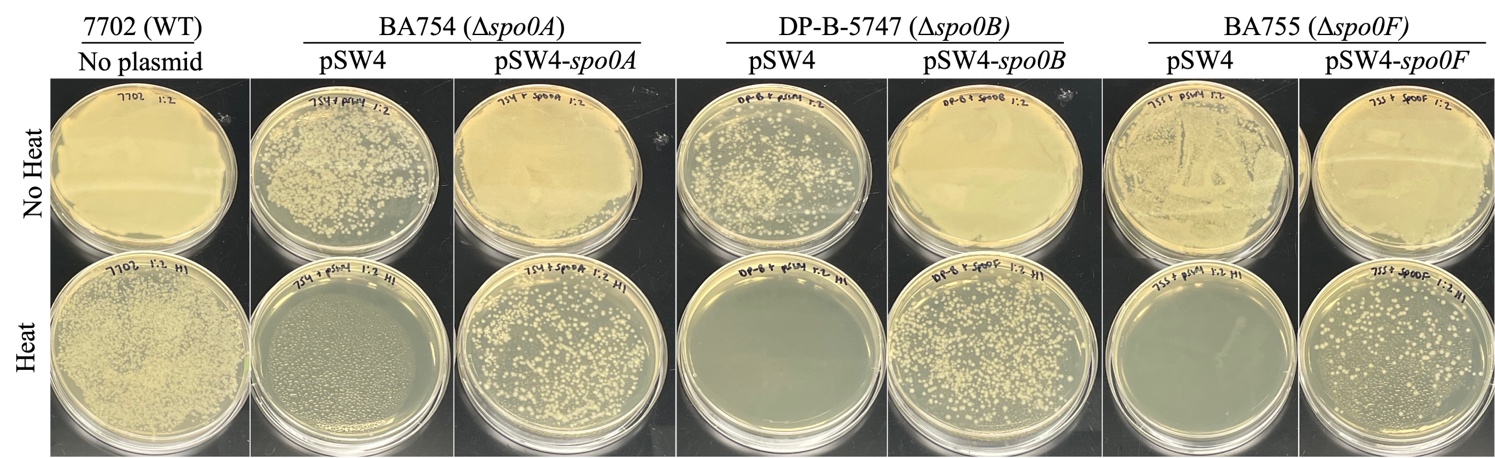


**Supplementary Figure 5.** Expression of *spo* genes in trans rescues sporulation phenotype in mutants. Cultures containing the empty pSW4 or pSW4 vector with the corresponding *spo0* gene were allowed to sporulate following the protocol described in the materials and methods section. Samples of equal volumes were aliquoted and exposed to heat (98°C) or room temperature incubation for 10 minutes. Samples were then plated on TSA, with the appearance of growth indicative of heat-resistant spores present in the initial culture.

| **Genome** | **Type** | **Gene** | **Description** | **Effect** | **AA change** | **WT7702** | **BA749** | **BA750** | **BA751** | **BA752** | **BA754** | **BA755** | **DP-B-5747** | **BAP350** |
| --- | --- | --- | --- | --- | --- | --- | --- | --- | --- | --- | --- | --- | --- | --- |
| Position |  |  |  |  |  |  | *∆BAS0566* | *∆sap* | ∆eag | *∆BAS1792* | *∆spo0A* | *∆spo0F* | *∆spoB* | *∆csaB* |
| 295979 | SNP | NGBLMGDH_00309 | hypothetical protein | Missense | Tyr401Asp | A | A | A | A | A | A | **C** | A | A |
| 615276 | DEL | smc_2 | chromosome partition protein Smc | Frameshift | Glu948fs | . | . | . | . | . | . | . | -tgaacatatacg | . |
| 935936 | SNP | rnz | ribonuclease Z | Missense | Gly84Glu | C | C | C | T | C | C | C | C | C |
| 994200 | SNP | noncoding | noncoding | Upstream gene | . | G | G | T | G | G | G | G | G | G |
| 1161006 | SNP | aspS | aspartate--tRNA ligase | Missense | Leu418Ile | G | G | G | G | G | G | G | T | G |
| 1411016 | SNP | msrC | free methionine-R-sulfoxide reductase | Silent | . | T | T | T | T | T | T | T | T | T |
| 1718679 | SNP | NGBLMGDH_01880 | hypothetical protein | Missense | Thr218Met | G | G | G | G | G | G | G | A | G |
| 2208563 | SNP | recR | recombination protein RecR | Missense | Pro6Ser | C | C | C | C | C | T | C | C | C |
| 2951785 | SNP | pbuO_3 | guanine/hypoxanthine permease PbuO | Missense | Ala390Glu | G | G | G | G | G | G | T | G | G |
| 2975790 | SNP | gloC_1 | hydroxyacylglutathione hydrolase GloC | Missense | Pro63Ser | C | C | C | T | C | C | C | C | C |
| 3789467 | SNP | slt | Soluble lytic murein transglycosylase | Missense | Gly121Val | G | G | G | G | G | G | G | T | G |

**Supplementary Table 1:** Summary report data following whole genome sequencing of *B. anthracis* Sterne mutant strains. Positions are provided based upon chromosomal location in the reference wild-type genome, *B. anthracis* strain 7702 (NCBI accession number CP110279). Residues that differ from the reference sequence are highlighted in red. SNP: single-nucleotide polymorphism. DEL: deletion. AA: amino acid.
